# Clinical Characteristics and Risk Factors for Cutaneous Manifestations Associated With Nemolizumab in Atopic Dermatitis: A Multicenter Retrospective Study in Japan

**DOI:** 10.1111/1346-8138.17877

**Published:** 2025-07-23

**Authors:** Wataru Sasaki, Ryo Saito, Kenta Suzuki, Daisuke Watanabe, Masako Minami‐Hori, Hirofumi Kamada, Hiroo Amano, Akihiko Uchiyama, Sei‐ichiro Motegi, Machiko Kamura, Kazunari Sugita, Noriko Kubota, Toshifumi Nomura, Maki Ozawa, Toshiya Takahashi, Takashi Yamakita, Kazumitsu Sugiura, Tetsuharu Ikegami, Ken Igawa, Yuka Kimura, Yoko Kataoka, Ryoichi Kamide, Masakazu Takahashi, Akio Tanaka, Mariko Sugawara‐Mikami

**Affiliations:** ^1^ Department of Dermatology Hiroshima University Hiroshima Japan; ^2^ Department of Dermatology Aichi Medical University Nagakute Aichi Japan; ^3^ Department of Dermatology Asahikawa City Hospital Asahikawa Hokkaido Japan; ^4^ Department of Dermatology Iwate Medical University Iwate Japan; ^5^ Department of Dermatology Gunma University Maebashi Gunma Japan; ^6^ Division of Dermatology, Department of Internal Medicine, Faculty of Medicine Saga University Saga Japan; ^7^ Department of Dermatology Tsukuba University Tsukuba Japan; ^8^ Department of Dermatology Tohoku University Graduate School of Medicine Sendai Miyagi Japan; ^9^ Department of Dermatology Fujita Health University, School of Medicine Toyoake Aichi Japan; ^10^ Department of Dermatology Dokkyo Medical University Tochigi Japan; ^11^ Department of Dermatology Osaka Habikino Medical Center Osaka Japan; ^12^ Department of Dermatology Hihuno Clinic Ningyocho Tokyo Japan; ^13^ Graduate School of Innovation and Technology Management Yamaguchi University Yamaguchi Japan; ^14^ West Yokohama Sugawara Dermatology Clinic Kanagawa Japan

**Keywords:** atopic dermatitis, cutaneous manifestations, immunological markers, nemolizumab, pruritus

## Abstract

Nemolizumab, an anti‐interleukin‐31 receptor A monoclonal antibody, has been approved in Japan for treating atopic dermatitis (AD)‐associated pruritus. While it is effective for itch control, nemolizumab‐associated cutaneous adverse events have been increasingly recognized, yet their clinical features remain poorly characterized. In this study, we aimed to investigate the incidence, clinical characteristics, and timing of cutaneous manifestations associated with nemolizumab treatment in patients with AD, and to explore potential associations with baseline disease severity and immunological parameters. We conducted a multicenter retrospective study involving 219 patients aged ≥ 13 years with AD who received nemolizumab at 13 institutions in Japan between August 2022 and February 2024. Cutaneous eruptions were classified into six categories based on clinical consensus. Patients who received fewer than three doses without developing skin reactions were excluded. Clinical and laboratory parameters were compared between patients with and without cutaneous manifestations. Cutaneous manifestations occurred in 88 patients (40.2%), most commonly within the first three doses. Erythema was the most frequent presentation (69.3%), and 62.5% of eruptions were non‐pruritic. No significant associations were observed between the occurrence of skin reactions and baseline eczema area and severity index scores, eosinophil counts, serum immunoglobulin E, or thymus and activation‐regulated chemokine levels. Two cases of bullous pemphigoid were identified. Despite topical corticosteroid treatment, nemolizumab therapy was discontinued in 42% of the patients affected. In conclusion, nemolizumab frequently induces early‐onset, morphologically distinct cutaneous eruptions that appear to be independent of baseline disease severity or biomarkers.

## 1 Introduction

Atopic dermatitis (AD) is a chronic inflammatory skin disease characterized by intense pruritus and eczematous lesions [1]. Recently, targeted therapies that modulate key pathogenic cytokines have expanded treatment options for AD. Interleukin‐31 (IL‐31) has been identified as a critical mediator of pruritus in AD, and therapies that block its receptor have become an established component of clinical practice [2, 3]. Nemolizumab, a humanized monoclonal antibody targeting the IL‐31 receptor A, was approved in Japan in 2022 for the treatment of pruritus associated with AD in patients aged 13 years and older [4]. While nemolizumab has demonstrated substantial efficacy in alleviating pruritus, clinical trials and post‐marketing surveillance have also reported cutaneous manifestations, including the appearance of new skin lesions and worsening of existing AD symptoms [5, 6, 7, 8].

Recent observations suggest that many cutaneous reactions associated with nemolizumab are not merely exacerbations of baseline AD but rather novel skin manifestations with distinct clinical characteristics [8, 9, 10, 11]. Furthermore, clinical trials in prurigo nodularis, a condition sharing overlapping inflammatory pathways with AD, have reported a lower incidence of such skin reactions compared with trials in AD [12]. These findings raise the possibility that patients with AD, particularly those with certain disease backgrounds, may be uniquely predisposed to nemolizumab‐associated cutaneous events. However, systematic characterization of these eruptions remains limited, and no standardized criteria exist for their evaluation. Furthermore, it remains unclear whether baseline clinical factors or immunological markers can predict the risk of developing such adverse events. Given the increasing use of nemolizumab in clinical practice, a better understanding of these skin manifestations is crucial for optimizing patient counseling, treatment decision‐making, and management strategies.

In this multicenter retrospective study, we aimed to characterize the clinical features and timing of cutaneous manifestations associated with nemolizumab treatment in patients with AD, and to investigate potential associations between baseline disease severity, immunological parameters, and the occurrence of these skin reactions.

## 2 Materials and Methods

### 2.1 Study Design and Setting

This multicenter retrospective study was conducted across 13 institutions in Japan that had administered nemolizumab to at least 10 patients each. The study period spanned between August 1, 2022, and February 29, 2024. The study protocol was approved by the institutional review boards of all participating institutions. Consent was obtained through an opt‐out process, in accordance with institutional and ethical guidelines. For patients whose clinical photographs were included in the publication, written informed consent was obtained. Patient data were anonymized prior to collection at Hiroshima University and subsequently analyzed at Hiroshima University and Yamaguchi University. The study design was approved by the Ethics Review Committee of Hiroshima University (E2024‐0004).

### 2.2 Patients

We included patients aged 13 years and older with a clinical diagnosis of AD who had received at least one dose of nemolizumab during the study period. Patients who developed cutaneous manifestations following nemolizumab administration were included, regardless of the number of doses received. Conversely, patients without cutaneous manifestations were excluded if they had received fewer than three doses, to minimize the risk of misclassifying late‐onset events. Patients with eruptions attributable to other identifiable causes, such as herpes zoster, were categorized as having “no cutaneous manifestations” and excluded from the main analysis.

Of the 219 cases analyzed in this study, 18 have been previously reported in the literature. Of these, 17 were included in studies that primarily focused on the therapeutic efficacy of nemolizumab [13]. In contrast, the present study aims to characterize the full spectrum of cutaneous manifestations, which have not been systematically investigated to date.

One additional case, featuring vesicular eruptions, has also been reported previously in isolation [9]; however, the current analysis is distinct in that it comprehensively evaluates a broader range of cutaneous manifestations potentially associated with nemolizumab administration.

### 2.3 Definition and Classification of Cutaneous Manifestations

**FIGURE 1:**
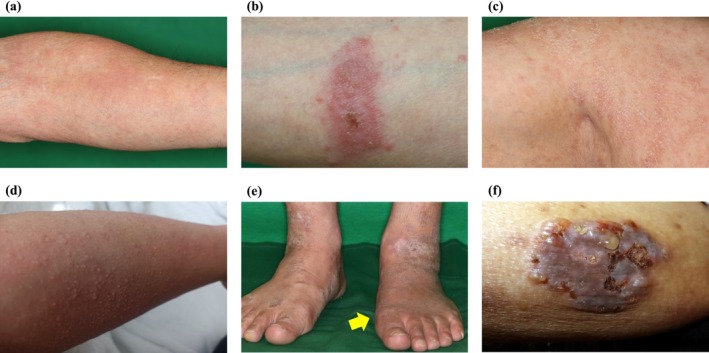
Representative clinical images of six distinct categories of cutaneous manifestations, as defined in this study: (a) erythema; (b) coin‐shaped red plaque with exudate; (c) dry skin/scaly lesions; (d) diffuse papules; (e) edema; and (f) vesicle.

In March 2024, dermatologists from the participating 13 institutions convened to discuss the clinical features of nemolizumab‐associated cutaneous manifestations based on their collective experience. Through this consensus process, six distinct categories of skin eruptions were established: erythema, coin‐shaped red plaques with exudates, dry skin or scaly lesions, papules, edema, and vesicles (Figure 1a–f). These categories were defined based on clinical morphology, and multiple types could occur concurrently or sequentially in the same patient. The term “worsening of AD” was intentionally excluded to differentiate newly emerging eruptions from exacerbations of baseline disease. In cases where manifestations could not be classified into the predefined categories, detailed clinical descriptions were recorded.

### 2.4 Data Collection

We retrospectively extracted clinical and laboratory data from the electronic medical records of all eligible patients. The collected information included demographic characteristics (age, sex, and history of allergic diseases), clinical features related to AD (Eczema Area and Severity Index [EASI] scores and the presence or absence of prurigo nodularis), and immunological parameters (peripheral blood eosinophil counts, serum immunoglobulin E [IgE] levels, and serum thymus and activation‐regulated chemokine [TARC] levels). Allergic diseases were defined based on documented comorbid diagnoses and included food allergy, allergic rhinitis, allergic conjunctivitis, asthma, and urticaria. Treatment‐related data were also collected, including the number of nemolizumab doses administered, the concomitant use and potency of topical corticosteroids, and details regarding treatment continuation or discontinuation. Regarding cutaneous manifestations, data on the morphology, timing of onset, affected body site(s) (e.g., head, face, neck, chest, abdomen), and presence or absence of associated pruritus were collected. In patients who developed multiple types of eruptions, each event was documented separately.

### 2.5 Outcome Measures

The primary outcome was the incidence of cutaneous manifestations associated with nemolizumab treatment. Secondary outcomes included the timing, morphology, and duration of eruptions; treatment continuation and discontinuation rates; and the association between baseline clinical or immunological factors and the occurrence of cutaneous manifestations.

### 2.6 Statistical Analysis

Statistical analyses were conducted using JMP Pro version 18.2.0 (SAS Institute Inc., Cary, NC, USA) and GraphPad Prism version 8 (GraphPad Software, San Diego, CA, USA). Group differences in the duration of cutaneous manifestations were assessed using the Mann–Whitney *U* test. Differences in the incidence of cutaneous manifestations across age groups (stratified by decades) were evaluated using the Chi‐squared test. Pearson's correlation coefficient was used to analyze the correlation between the median age of each group and the incidence rate of cutaneous manifestations. To ensure analytical reliability, age groups comprising fewer than five individuals were excluded; accordingly, the 90‐ to 99‐year age group (*n* = 3) was not included in the analysis. All other comparisons were performed using Student's *t*‐test. All statistical analyses were performed on untransformed data, and a two‐sided *p*‐value of < 0.05 was considered indicative of statistical significance.

Data visualization, including tables and graphs, was performed using Microsoft Excel (Microsoft Corp., Redmond, WA, USA) and GraphPad Prism version 8.

## 3 Results

### 3.1 Patient Characteristics

We analyzed a cohort of 219 patients with AD who received nemolizumab at 13 institutions in Japan. The study population consisted of 133 men (60.7%) and 86 women (39.3%), with a median age of 51 years (interquartile range [IQR], 26–75) (Table 1). Median EASI score was 17.7 (IQR, 11.7–25.3), indicating that nemolizumab was predominantly administered to patients with moderate to severe AD. Notably, 94 patients (42.9%) had a history of systemic immunomodulatory therapy prior to nemolizumab initiation, including corticosteroids, cyclosporine, Janus kinase (JAK) inhibitors, or dupilumab. This suggests that nearly half had previously experienced inadequate disease control with topical therapy alone.

**TABLE 1:** Baseline characteristics of the study patients.

Male sex, *n* (%)	133 (60.7)
Age (years), median (Q1–Q3)	51 (26–75)
EASI score, median (Q1–Q3)	17.7 (11.7–25.3)
Pruritus NRS score, median (Q1–Q3)	8 (6–8)
Eosinophil counts (/μL), median (Q1–Q3)	365.7 (200.5–704.2)
IgE (IU/mL) median (Q1–Q3)	1033.4 (199.1–3918.5)
TARC (pg/mL), median (Q1–Q3)	976.0 (478.3–2429.8)
Past systemic treatment, *n* (%)	
Oral prednisolone	33 (15.1)
Oral Cyclosporine A	30 (13.7)
Oral JAK inhibitor	25 (11.4)
Dupilumab	24 (11.0)
Any systemic treatment	94 (42.9)
Allergic disease at baseline, *n* (%)	
Seasonal allergy	7 (3.2)
Allergic rhinitis	17 (7.8)
Allergic Conjunctivitis	6 (2.7)
Food allergy	3 (1.4)
Asthma	36 (16.4)
Urticaria	6 (2.7)
Any allergic disease	57 (26.0)
Type of atopic dermatitis, *n* (%)	
Nodular prurigo	93 (42.5)
Non nodular prurigo	126 (57.5)

*Note:* Data are presented as numbers (%) for categorical variables and as median (interquartile range, Q1–Q3) for continuous variables. Laboratory data, including eosinophil counts, serum immunoglobulin E (IgE) levels, and thymus and activation‐regulated chemokine (TARC) levels, were obtained from the most recent assessments before initiating nemolizumab treatment. Previous systemic treatments included oral prednisolone, cyclosporine A, Janus kinase (JAK) inhibitors, and subcutaneous dupilumab. Baseline allergic comorbidities were recorded based on patient history. The type of atopic dermatitis was classified as nodular prurigo or non‐nodular prurigo according to clinical diagnosis.

### 3.2 Incidence and Types of Cutaneous Manifestations

Cutaneous manifestations that developed after nemolizumab initiation were recorded and classified. In cases where multiple distinct eruption types occurred in the same patient, each type was counted separately. Cutaneous manifestations were observed in 88 patients (40.2%), which were systematically categorized into six morphological types: erythema, coin‐shaped plaques with exudate, dry/scaly lesions, papules, edema, and vesicles (Table 2, Figure 1). The presence of cutaneous eruptions did not significantly differ between male and female patients, and the pattern of eruption types showed no sex‐related differences (data not shown). Erythema was the most frequently reported (69.3%), while vesicular eruptions were relatively uncommon (6.8%). Two cases of vesicular eruption were diagnosed as bullous pemphigoid (BP) based on serological and histopathological criteria. Other types of cutaneous manifestations included one case each of urticaria and hair loss, in addition to the six defined categories.

**TABLE 2:** Characteristics of the cutaneous manifestations following nemolizumab treatment.

Cutaneous manifestations, *n* (%)	88 (40.2)
Erythema	61 (69.3)
Coin‐shaped red plaques with exudate	37 (42.0)
Dry skin/scaly lesions	26 (29.5)
Papules	19 (21.6)
Edema	9 (10.2)
Vesicles	6 (6.8)
Others	2 (2.3)
Itch (pruritus) of eruption, *n* (%)	
Present	33 (37.5)
Absent	55 (62.5)

*Note:* Types of skin eruptions were classified based on clinical appearance. For patients who developed multiple types of eruptions, each event was documented separately. Recurrent eruptions occurring at different time points were also counted individually. Data are presented as number (%). The presence or absence of pruritus associated with the eruptions was also recorded.

This classification scheme facilitated a structured evaluation of clinically distinct eruption patterns potentially related to nemolizumab.

### 3.3 Timing of Initial Onset

**FIGURE 2:**
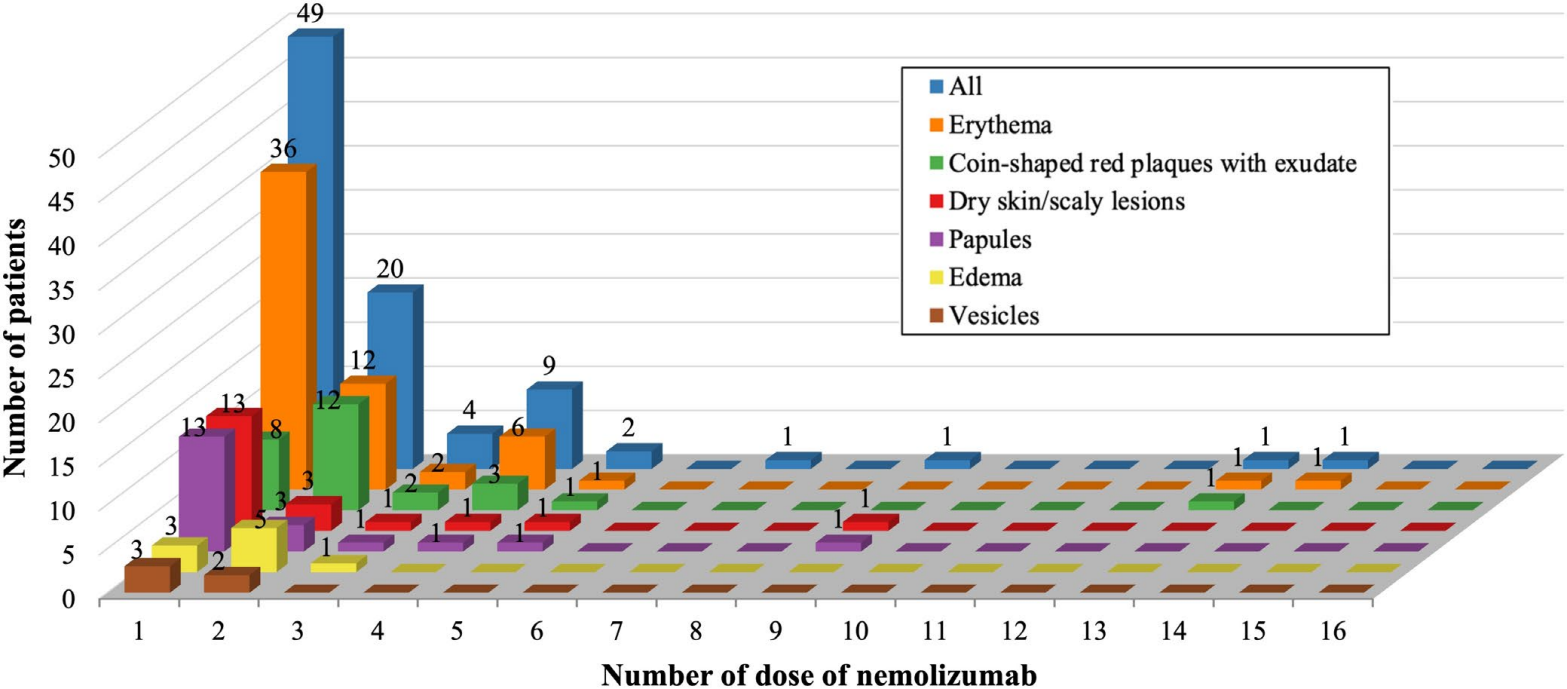
Timing of the initial appearance of cutaneous manifestations following nemolizumab treatment. The vertical axis represents the number of cases, and the horizontal axis indicates the number of nemolizumab doses administered. Blue bars represent the total number of eruptions, and each eruption type is color‐coded: orange for erythema, green for coin‐shaped red plaques with exudates, red for dry skin/scaly lesions, purple for papules, yellow for edema, and brown for vesicles. No new eruptions were observed beyond the 16th dose.

The timing of initial onset of cutaneous eruptions is illustrated in a bar graph according to eruption type (Figure 2). Among those who developed eruptions, approximately 80% did so within the first three doses, with the first dose alone accounting for a large proportion of early events. Each eruption type showed characteristic timing, with erythema and dry lesions occurring earlier, while coin‐shaped plaques more commonly arose after the second dose. Notably, the frequency of initial eruptions markedly declined after the sixth dose, with no cases of edema or vesicles reported beyond this point.

These temporal trends help clinicians anticipate the typical treatment window during which adverse cutaneous reactions are most likely to occur.

### 3.4 Anatomical Site of First Cutaneous Eruption

The anatomical sites and frequencies of the first appearance of cutaneous eruptions are summarized in Table 3. All types of eruptions were included regardless of morphology, and multiple sites per patient were counted separately. The forearms were the most commonly affected initial site (61.4%), followed by the back (51.1%), and both the upper arms and lower legs (48.9%). In contrast, eruptions on the hands, feet, and buttocks were less frequently observed. These distribution patterns may assist clinicians in identifying typical sites of early cutaneous involvement during nemolizumab treatment. The distribution of baseline AD lesions was not recorded in this study, and it was therefore not assessed whether the initial eruptions appeared at sites previously affected by AD.

**TABLE 3:** Anatomical distribution of the first appearance of cutaneous manifestations.

Initial cutaneous manifestations, *n* (%)	88 (40.2)
Head	10 (11.4)
Face	29 (33.0)
Neck	14 (15.9)
Chest	34 (38.6)
Abdomen	38 (43.2)
Back	45 (51.1)
Upper arm	43 (48.9)
Forearm	54 (61.4)
Hand	18 (20.5)
Buttocks	15 (17.0)
Thigh	38 (43.2)
Lower leg	43 (48.9)
Foot	16 (18.2)

*Note:* Anatomical distribution of the first appearance of skin manifestations following initiation of nemolizumab treatment. All lesion types were included regardless of morphology, and lesions occurring in multiple anatomical sites were counted separately. The figure presents the number of lesion events and the proportion of patients with lesions at each site.

### 3.5 Treatment Continuation and Topical Therapy

**FIGURE 3:**
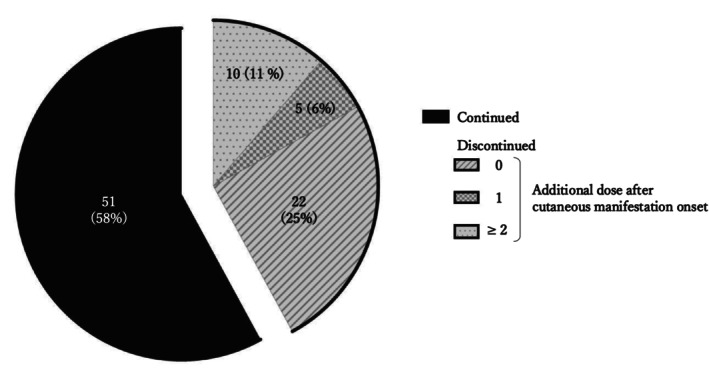
Treatment status of patients after the onset of cutaneous manifestations. The pie chart illustrates the proportion of patients who continued or discontinued nemolizumab after developing cutaneous manifestations, as well as the timing of treatment discontinuation relative to eruption onset. The black segment represents patients who continued treatment, while the gray area indicates those who discontinued. Within the gray area, hatched, shaded, and dotted segments represent patients who discontinued treatment without additional dosing, after one additional dose, and after two or more additional doses, respectively.

Figure 3 illustrates whether nemolizumab treatment was continued or discontinued following the onset of cutaneous eruptions and, in discontinued cases, how many additional doses were administered after the onset of cutaneous eruptions and before treatment discontinuation. Despite the occurrence of cutaneous eruptions, 51 patients (58.0%) continued nemolizumab treatment. Among the 37 patients who discontinued, most (59.5%) did so immediately after eruption onset. All patients received topical corticosteroids, with strong or very strong potency agents being used in 94% of cases (data not shown).

### 3.6 Duration of Eruptions

**FIGURE 4:**
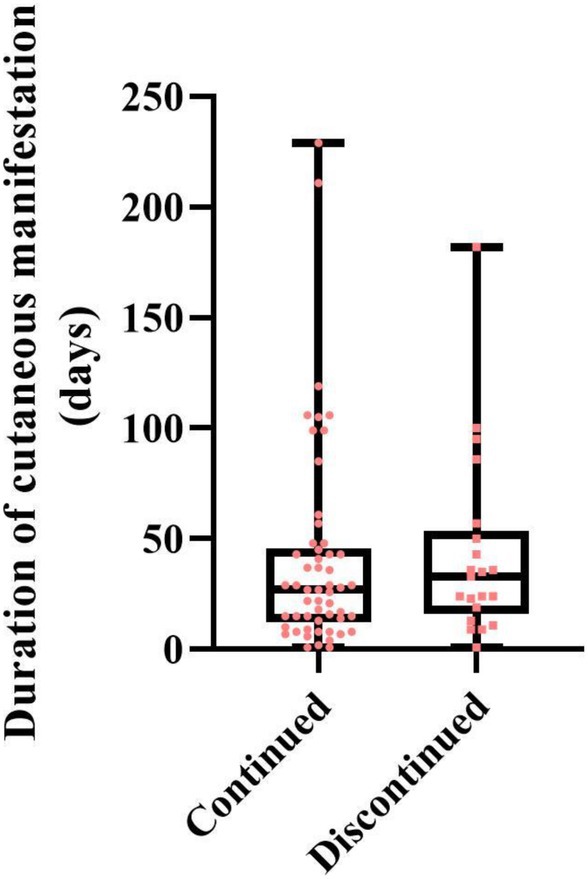
Duration of cutaneous manifestations in patients who continued or discontinued nemolizumab treatment. Data were available for 75 patients. The median duration of symptoms was 33 days in patients who discontinued treatment and 27 days in those who continued, with no statistically significant difference (*p* = 0.38, Mann–Whitney *U* test). In the box plots, boxes represent the median and interquartile range (IQR), whiskers indicate the minimum and maximum values, and individual patient data points are plotted separately.

Among patients who developed cutaneous eruptions, the resolution of skin symptoms could be clearly determined in 75 cases, and the duration of eruptions was compared between patients who continued nemolizumab and those who discontinued following eruption onset (Figure 4). The median duration of symptoms was 33 days (IQR, 19.0–50.0) for those who discontinued treatment and 27 days (IQR, 13.25–44.5) for those who continued, without a statistically significant difference (*p* = 0.38, Mann–Whitney *U* test).

### 3.7 Baseline Clinical and Laboratory Parameters

**FIGURE 5:**
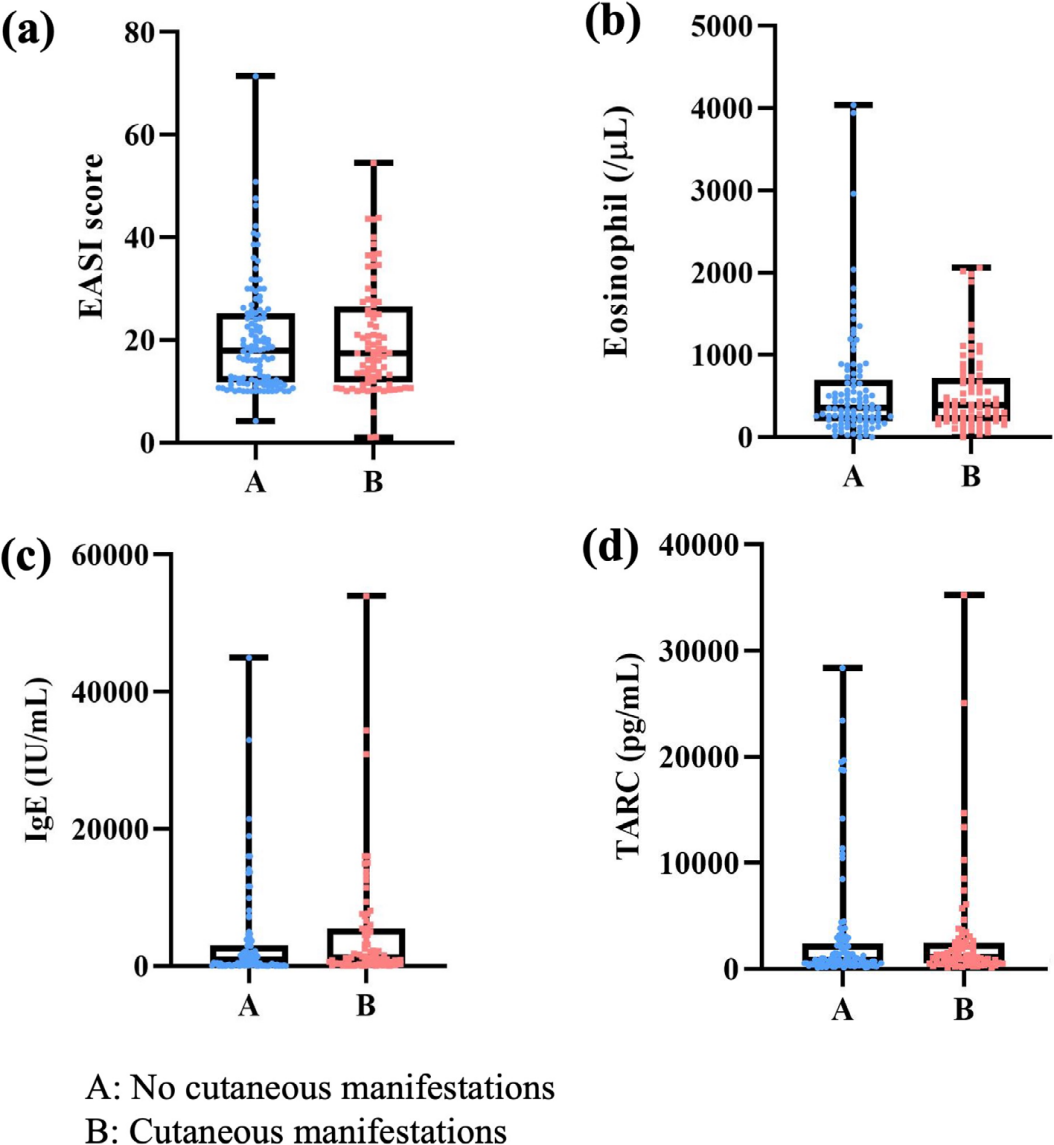
Relationship between atopic dermatitis severity and cutaneous manifestations. Box plots comparing (A) patients without cutaneous manifestations and (B) patients with cutaneous manifestations in terms of (a) Eczema Area and Severity Index (EASI) score, (b) eosinophil count, (c) immunoglobulin E (IgE) levels, and (d) thymus and activation‐regulated chemokine (TARC) levels. In each box plot, boxes represent the median and interquartile range (IQR), whiskers indicate the minimum and maximum values, and individual patient data points are plotted separately. No statistically significant differences were observed between groups (*p* > 0.05, Student's *t*‐tests). [Correction added on 26 October 2025 after first online publication: In Fig 5(d), the unit of the y‐axis has been updated as “TARC (pg/mL)” instead of “TARC (pg/μL)”.]

**FIGURE 6:**
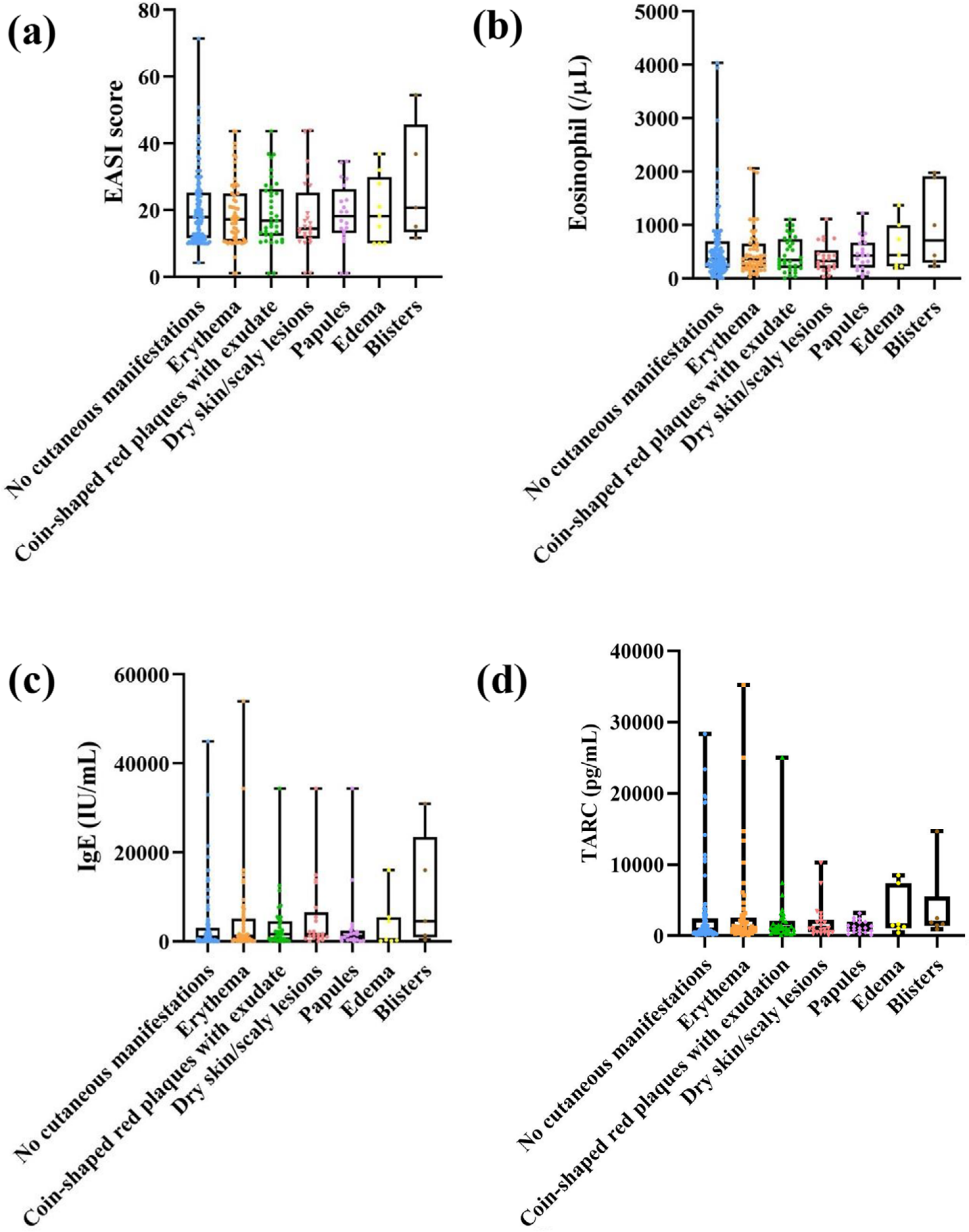
Relationship between baseline atopic dermatitis severity and laboratory markers among six types of cutaneous manifestations. Box plots showing the comparison of baseline clinical and laboratory parameters among six distinct categories of skin eruptions independently defined in this study: erythema, coin‐shaped red plaques with exudates, dry skin/scaly lesions, papules, edema, and vesicles. The parameters evaluated were (a) Eczema Area and Severity Index (EASI) score (clinical assessment), (b) eosinophil count, (c) immunoglobulin E (IgE) levels, and (d) thymus and activation‐regulated chemokine (TARC) levels (laboratory markers). In each box plot, boxes represent the median and interquartile range (IQR), whiskers indicate the minimum and maximum values, and individual patient data points are plotted separately. No statistically significant differences were observed among eruption types (*p* > 0.05, Student's *t*‐tests). [Correction added on 26 October 2025 after first online publication: In Fig 6(d), the unit of the y‐axis has been updated as “TARC (pg/mL)” instead of “TARC (pg/μL)”.]

To explore predictive factors, we compared baseline disease severity and immunological markers between patients with and without eruptions. No significant differences were found in EASI scores, eosinophil counts, serum IgE, or TARC levels (Figure 5a–d). Furthermore, no clear associations were noted between laboratory markers and specific eruption types (Figure 6a–d). These findings suggest that baseline disease severity does not appear to predict the likelihood or morphology of nemolizumab‐related skin reactions.

### 3.8 Demographic and Clinical Correlates

**FIGURE 7:**
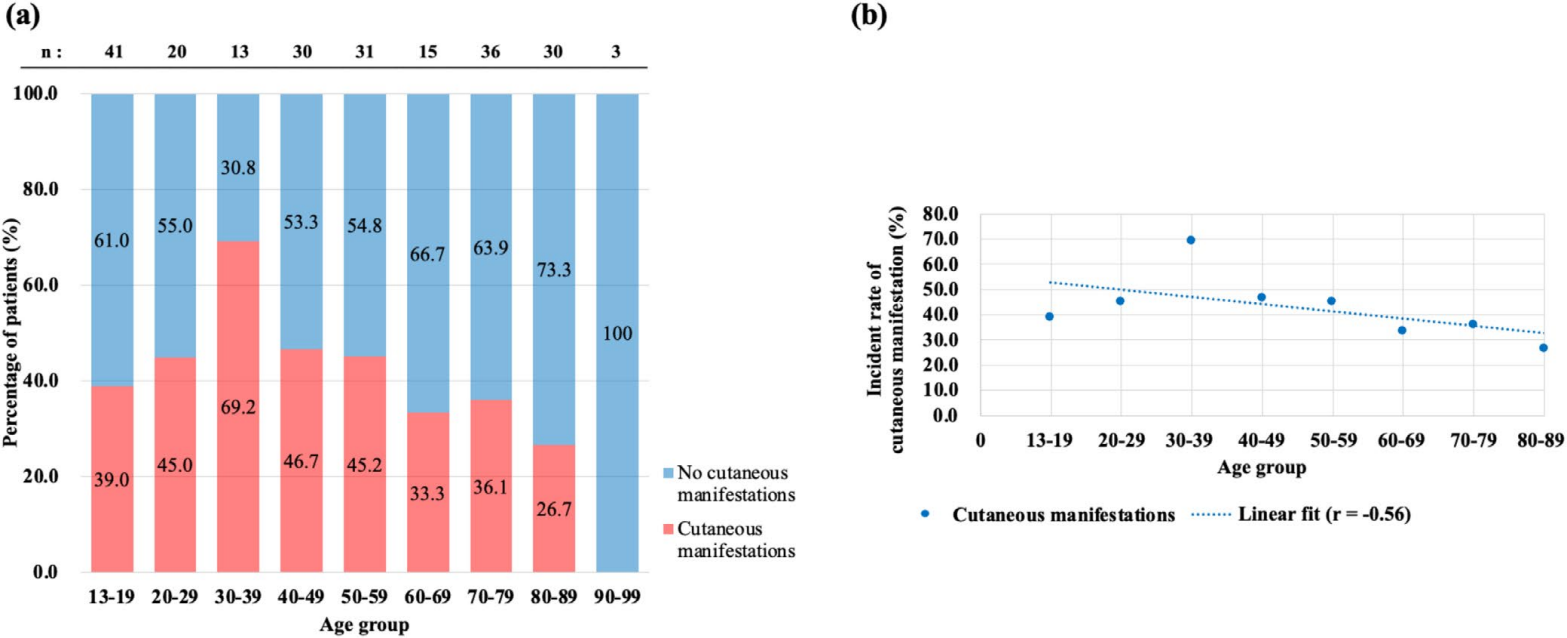
Relationship between age and incidence of cutaneous manifestations during nemolizumab treatment. (a) The proportion of patients who developed or did not develop cutaneous manifestations across different age groups stratified by decade. The vertical axis represents the percentage of patients, and the horizontal axis indicates the age groups. The number above each bar indicates the total number of patients in each age group. (b) Correlation between the median age of each group and the incidence rate of skin eruptions, showing a moderate inverse correlation (Pearson's *r* = −0.56). Patients aged 90–99 years were excluded from the correlation analysis due to the small sample size (*n* = 3).

**FIGURE 8:**
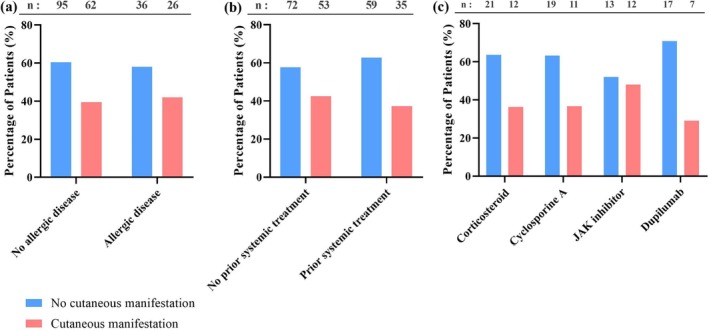
Relationship between baseline clinical characteristics and the incidence of cutaneous manifestations during nemolizumab treatment. (a) Proportion of patients with and without cutaneous manifestations according to the presence of comorbid allergic conditions (e.g., food allergy, allergic rhinitis, allergic conjunctivitis, asthma, and urticaria). (b) Proportion of patients with and without cutaneous manifestations according to the history of systemic treatment prior to nemolizumab initiation. (c) Proportion of patients with and without cutaneous manifestations according to the type of prior systemic treatment, including oral corticosteroids, oral cyclosporine A, oral Janus kinase (JAK) inhibitors, and subcutaneous dupilumab. No statistically significant associations were observed in any of the comparisons (*p* > 0.05, Student's *t*‐tests).

To evaluate the relationship between age and the incidence of cutaneous manifestations, the proportion of patients with eruptions was visualized by age (Figure 7a). No statistically significant association was observed between age group and the incidence of cutaneous manifestations (*p* = 0.234, Chi‐squared test). The incidence rates remained relatively uniform across age groups (ranging from 30% to 50%); however, a moderate inverse correlation was identified between the median age of each group and the incidence rate (Pearson's *r* = −0.56, *p* = 0.16) (Figure 7b). In the correlation analysis, the 90‐ to 99‐year age group was excluded to ensure analytical reliability because of the small sample size (*n* = 3). Comorbid allergic conditions, the presence of prurigo nodularis, and a history of systemic treatment prior to nemolizumab initiation were not significantly associated with the risk of cutaneous eruptions (Figure 8a–c). This reinforces the observation that demographic or clinical background factors may not be reliable indicators of susceptibility to these skin events.

## 4 Discussion

In this multicenter retrospective study, approximately 40% of patients with AD treated with nemolizumab developed cutaneous manifestations. These eruptions predominantly occurred within the first three doses and showed consistent clinical patterns, most commonly presenting as erythema, followed by coin‐shaped plaques with exudate. The initial eruptions most frequently appeared on the forearms, back, upper arms, and lower legs, whereas involvement of the hands, feet, and buttocks was less common. Notably, more than 60% of the eruptions were non‐pruritic, and there were no associations with baseline disease severity, prior systemic treatments, or Type 2 inflammatory markers such as eosinophils, IgE, or TARC levels [14, 15, 16].

The clinical profile of these eruptions (early‐onset, morphologically distinct, and largely non‐pruritic) suggests that they are not simply flares of underlying AD but may represent a phenomenon induced by IL‐31 receptor blockade. This interpretation is further supported by the absence of similar findings in studies of IL‐4/IL‐13 inhibitors such as dupilumab [17, 18], tralokinumab [19, 20], or lebrikizumab [21], indicating a mechanistic specificity to IL‐31 pathway inhibition. Histopathological confirmation was not obtained in this study; however, the clinical features were consistent with spongiotic dermatitis, indicating a type of eczematous inflammation possibly driven by altered immune regulation after IL‐31 inhibition.

IL‐31 plays a pivotal role in neuroimmune communication, and its blockade may disrupt feedback loops that regulate T helper 2 cytokine responses [22, 23, 24]. Recent studies have suggested that IL‐31 promotes calcitonin gene‐related peptide release from sensory neurons, which suppresses IL‐13 production by CD4^+^ T cells. Inhibition of IL‐31 signaling could thus remove this regulatory constraint, leading to enhanced IL‐13‐driven inflammation and subsequent eczematous skin changes [25].

Among the patients who developed vesicular eruptions, two cases were diagnosed with BP, supported by elevated anti‐BP180 antibody levels and histopathological confirmation in one case. These findings suggest that nemolizumab may trigger the clinical onset of autoimmune skin diseases in patients with preexisting subclinical autoantibody production or latent immunological abnormalities. While such occurrences are rare, they highlight the need for vigilance, particularly in older patients or those with a history suggestive of autoimmune susceptibility.

Despite the relatively high incidence of cutaneous reactions, 58% of patients were able to continue nemolizumab treatment with the use of high‐potency topical corticosteroids. When eruption duration was compared between those who continued treatment and those who discontinued, no significant difference was observed. This suggests that stopping nemolizumab does not necessarily lead to faster improvement of skin symptoms.

One possible explanation lies in the drug's pharmacological characteristics. The pharmacokinetic profile of nemolizumab is characterized by slow absorption, with peak serum concentrations observed 4.5–9.2 days after administration, and a long terminal half‐life ranging from 12.6 to 16.5 days [26]. Notably, nemolizumab has also been shown to exert sustained biological effects even after treatment discontinuation, particularly in maintaining antipruritic efficacy [8, 27, 28, 29, 30]. These prolonged pharmacodynamic effects are thought to result from continued modulation of IL‐31‐mediated neural and immune pathways. While this persistence is beneficial for itch control, it may also contribute to the continued presence of cutaneous adverse events after drug cessation.

From a clinical perspective, these findings carry important implications. In patients who develop mild eruptions, nemolizumab therapy can often be continued with appropriate escalation of topical corticosteroid treatment. Conversely, in patients with severe eruptions, discontinuation of nemolizumab is often unavoidable. However, clinicians should be aware that even after stopping the drug, immediate resolution of skin symptoms should not be expected. This understanding allows for more realistic patient counseling and encourages adherence to intensified topical regimens, rather than prematurely discontinuing nemolizumab treatment based solely on the assumption of rapid improvement.

A practical management approach would involve early identification and classification of eruption morphology, timely intensification of topical therapy, and close monitoring during the initial treatment period, particularly through the third dose when most reactions tend to occur.

Notably, we were unable to identify any baseline characteristics that could predict the occurrence of nemolizumab‐associated cutaneous manifestations. Neither demographic factors, such as age or sex, nor clinical features, such as a history of allergic diseases or the presence of prurigo nodularis, were associated with an increased risk. This underscores the need for future prospective studies to identify immunological or genetic markers that may predispose certain patients to develop these reactions.

This study has some limitations. First, cutaneous eruptions were classified based solely on clinical appearance, without histopathological or immunological confirmation. Second, because of the retrospective design, serial data on biomarkers or skin conditions over time could not be collected, limiting insights into the dynamic course of these adverse events. Third, patient‐reported outcomes such as quality of life or subjective itch intensity were not systematically assessed, which may have underestimated the clinical burden of these manifestations. Fourth, the multi‐institutional setting could have introduced variability in the assessment and reporting of skin reactions. Finally, we were unable to investigate potential immunogenetic factors, such as HLA type or autoantibody status, that may predispose patients to more severe cutaneous adverse events.

In conclusion, nemolizumab‐induced cutaneous manifestations are relatively common but generally manageable events, typically arising early in treatment and unrelated to baseline disease severity. Mild cases can often be managed without discontinuing therapy, whereas severe eruptions may necessitate cessation of treatment, with the expectation that skin symptoms may persist for some time even after discontinuation. Awareness of these clinical features can help optimize the management and counseling of patients receiving nemolizumab.

## Ethics Statement

The study protocol was approved by the institutional review boards of all participating institutions, including the Ethics Review Committee of Hiroshima University (E2024‐0004).

## Consent

Consent was obtained through an opt‐out process, in accordance with institutional and ethical guidelines. For patients whose clinical photographs were included in the publication, written informed consent was obtained.

## Conflicts of Interest

K. Sugiura, S.M., D.W., H.A., T.N., and A.T. have received research grants from Maruho. K. Sugita has received research grants from Maruho and Torii Pharma, and Y. Kimura and Y. Kataoka have received research grants from Sanofi, AbbVie, Amgen, Eli Lilly Japan, LEO Pharma, Maruho, Otsuka Pharmaceutical, Taiho Pharmaceutical, and Pfizer. S.M., D.W., and A.T. have received consulting fees from Maruho, while S.M. has also received consulting fees from AbbVie and LEO Pharma, and A.T. from Torii Pharma, Sanofi, and Otsuka Pharma. K. Sugiura, S.M., D.W., K.I., H.A., T.N., M.O., Y. Kataoka, K. Sugita, A.T., and M.S.‐M. have received lecturer honoraria from Maruho. Additionally, K. Sugiura, M.M.‐H., S.M., K.I., H.A., T.N., M.O., Y. Kataoka, K. Sugita, and A.T. have received honoraria from Sanofi. K. Sugiura, S.M., K.I., H.A., T.N., M.O., Y. Kataoka, K. Sugita, and A.T. have received honoraria from AbbVie. Similarly, K. Sugiura, S.M., K.I., H.A., T.N., M.O., Y. Kataoka, K. Sugita, and A.T. have received honoraria from Eli Lilly, and K. Sugiura, S.M., K.I., H.A., T.N., M.O., and Y. Kataoka have received honoraria from LEO Pharma. K. Sugiura, S.M., K.I., H.A., T.N., M.O., Y. Kataoka, K. Sugita, and A.T. have received lecture honoraria from Pfizer, while S.M., K.I., T.N., M.O., K.S., Y.K., A.T., and A.T. have received lecture honoraria from Otsuka Pharma. Additionally, K. Sugiura, S.M., T.N., M.O., K. Sugita, and A.T. have received lecture honoraria from Torii Pharma, and K. Sugiura and Y. Kataoka have received lecture honoraria from Taiho Pharma. D.W. has received lecture honoraria from GSK.

## Data Availability

The data that support the findings of this study are available on request from the corresponding author. The data are not publicly available due to privacy or ethical restrictions.
